# Protective effects of an Fc-engineered PD-L1 fusion protein in a spontaneous abortion model and a Th17 cell–induced pregnancy loss model

**DOI:** 10.3389/fphar.2025.1703805

**Published:** 2025-12-09

**Authors:** Guangyao Li, Tiantian Wang, Dan Tan, Yamin Jin, Xuemei Zhou, Hongru Ai, Xinya Zheng, Xiaochen Ren, Shi Hu, Changhai Lei, Wenyan Fu

**Affiliations:** 1 Department of Biomedical Engineering, College of Basic Medical Sciences, Second Military Medical University, Shanghai, China; 2 Department of Assisted Reproduction, Shanghai Ninth People’s Hospital, Shanghai Jiao Tong University School of Medicine, Shanghai, China; 3 Department of Biophysics, College of Basic Medical Sciences, Second Military Medical University, Shanghai, China

**Keywords:** programmed cell death 1 receptor, PD-L1, immunoglobulin Fc fragments, pregnancy complications, immunologic, T-lymphocytes, Th17, immunetolerance

## Abstract

The binding of programmed death-ligand 1 (PD-L1) with its receptor PD-1 has been proven to negatively regulate immune responses. Here, we assessed the therapeutic effects of Fc-fused PD-L1 protein on foetomaternal tolerance using a murine spontaneous abortion model and a Th17 cell-induced abortion model. Fc-engineered recombinant PD-L1-Fc showed negligible cytotoxicity to PD1-positive cells. The abortion rates in the CBA/J × DBA/2 mouse model were significantly ameliorated after PD-L1-Fc treatment. Additionally, PD-L1-Fc administration decreased the expression of interleukin (IL)-17A and diminished the frequency of IL-17A-producing CD4^+^ T cells in the decidua of treated mice. The foetomaternal protective effect of PD-L1-Fc was also observed in a Th17 cell transfer-induced abortion mouse model. These results indicate that PD-L1-Fc may provide a novel therapeutic strategy to treat spontaneous abortion involving immune factors.

## Introduction

The binding of PD-1 to PD-L1 delivers a suppressive signal to T cells, curtailing cytokine production and proliferation (Freeman et al., 2000). Once viral infection occurs, PD-L1 expression by cells can protect against cytotoxic T cell CTL killing and induce a regulatory mechanism that potentially ameliorates chronic immune responses (Keir et al., 2007).

Two mechanisms of peripheral tolerance were represented by the PD-1/PD-L1 pathway to defend against potentially pathogenic effector T cells. First, the pathway promotes Treg development and function. Second, it shows a direct inhibitory effect on pathogenic effector T cells. Spontaneous autoimmunity diseases, such as lupus-like glomerulonephritis (Nishimura et al., 1999) and cardiomyopathy (Nishimura et al., 2001), were observed in PD-1-deficient mice. Facing the maternal bloodstream in the uterus, the outer surface of syncytiotrophoblasts on chorionic villi in the placenta expresses high levels of PD-L1. Low PD-L1 expression was detected on the inner aspect of the syncytiotrophoblasts facing chorionic villous stroma and on cytotrophoblasts facing foetal blood vessels (Veras et al., 2017; Holets et al., 2006; Petroff et al., 2003). Although it is semiallograft to the maternal immune system with the expression of both allogeneic paternal antigen and autogenic maternal antigen, the foetus is not rejected by the maternal immune system. This paradox may be partly explained by the high level of PD-L1 on the placenta; similar to cancer cells, expressing PD-L1 can render cytotoxic T lymphocytes (CTLs) inactivated or nonfunctional through engagement of the inhibitory receptor PD-1, and the placenta expresses PD-L1 to escape T-cell attachment and induce immune tolerance to the foetus via PD-1 on T cells. Moreover, experimental evidence has established that PD-L1 is a key player in maternal-foetal tolerance. A mouse pregnancy model revealed that foetal loss is induced by direct blockade of the PD1/PDL1 pathway by an anti-PD-L1 monoclonal antibody during pregnancy (Guleria et al., 2005). Moreover, PD-L1 blockade results in a reduction in litter size and number and an increase in embryo resorption in mice and the failure of foetal-maternal tolerance with Treg deficiency and hyperactivity of Th17 cells (D’Addio et al., 2011). Interestingly, the level of the circulating soluble form of PD-L1, which increases when the expression of PD-L1 is unregulated, was notably elevated in pregnant women compared with that in nonpregnant women (Okuyama et al., 2019), further highlighting the importance of this signalling pathway. Elevated circulating soluble PD-L1 during healthy pregnancy has been correlated with immune tolerance, whereas dysregulated PD-1/PD-L1 signaling has been associated with recurrent spontaneous abortion and preeclampsia. Although no PD-L1-Fc–based therapeutics have been clinically evaluated in reproductive disorders, these observations highlight the translational relevance of modulating PD-1 signaling in human pregnancy complications.

Although wild-type PD-L1-Fc has shown therapeutic potential in autoimmune models such as colitis and arthritis, whether Fc-engineered, ADCC-silent PD-L1-Fc can modulate the unique immune microenvironment at the maternal–fetal interface remains unknown. Pregnancy tolerance involves distinct immunological processes compared with systemic autoimmunity, including uterine-specific Th17 restraint, trophoblast–immune interactions, and fine regulation of PD-1–expressing decidual leukocytes. These pregnancy-specific features provide a compelling rationale to investigate PD-L1-Fc in abortion-prone models.

To improve the *in vivo* stability of therapeutic proteins, a well-established genetic preparation method involves fusing the crystallisable fragment (Fc) of human immunoglobulin G (IgG) with recombinant receptor extracellular domains (Jafari et al., 2017). Previous reports have shown that soluble PD-L1-Fc protein treatment diminished the severity of mouse models of collagen-induced arthritis (Wang et al., 2011) and T-cell-induced chronic colitis (Song et al., 2015). However, whether PD-L1-Fc protein has a protective role in experimental abortion-prone animal models has not been explored. In this study, we first generated and characterised a recombinant Fc-engineered PD-L1-Fc protein. In contrast to previous studies using wild-type PD-L1-Fc in autoimmune or inflammatory models, the present study investigates whether an Fc-engineered, ADCC-deficient (LALA-PG) PD-L1-Fc can promote fetomaternal tolerance while avoiding Fc-mediated cytotoxicity. Our data showed that PD-L1-Fc administration ameliorated spontaneous abortion in a DBA/2J × CBA/J mouse model. The therapeutic effect of PD-L1-Fc in a mouse model was related to a decreased frequency of Th17 cells and increased production of IL-10-producing CD4 T cells. Additionally, PD-L1-Fc efficiently prevented foetal rejection in a Th17 cell adoptive transfer mouse model of abortion, providing evidence that PD-L1-Fc is particularly suitable for treating Th17-dependent abortion. We hypothesized that an Fc-engineered, non-cytolytic PD-L1-Fc fusion protein would enhance fetomaternal immune tolerance by suppressing Th17-mediated inflammation while avoiding depletion of PD-1–expressing immune subsets. We further anticipated that PD-L1-Fc would reduce fetal resorption in both spontaneous and Th17-driven abortion models.

## Materials and methods

### Fusion protein preparation

Ligation of the DNA sequences of the extracellular domains (ECDs) of murine PD-L1 to the DNA sequence of the wild-type or mutant Fc segment of murine IgG2a was conducted to generate recombinant fusion plasmids. Integrated DNA Technologies was used to generate mutations. The pcDNA3.4 vector and FreeStyle 293 expression system (Invitrogen) were used for fusion protein expression. Cell culture supernatants were harvested to obtain further purified fusion proteins using protein A-Sepharose. The concentrations and purity of the fusion proteins were determined by measuring the UV absorbance at a wavelength of 280 nm and by polyacrylamide gel electrophoresis, respectively.

### *In vitro* ADCC assay

Target cells (1 × 10^6^) were labelled with radioactive ^51^Cr (50 μCi) for 1 h at 37 °C. The labelled target cells were plated in 96-well plates at 5,000 cells per well in a volume of 100 μL. Next, effector cells were added according to different E:T ratios with different drugs. The cell supernatants (30 μL from each well) were collected after 4 h of incubation at 37 °C and transferred to a LumaPlate filter, which was allowed to dry overnight. A β-emission-reading liquid scintillation counter was used to measure the radioactivity released into the culture medium. The percentage of specific lysis was calculated as follows: (sample counts–spontaneous counts)/(maximum counts–spontaneous counts) × 100.

### Affinity measurement

Immobilised anti-human Fc polyclonal antibody (Jackson ImmunoResearch; 109-005-008) on a CM5 chip (∼150 RU) was used to capture the fusion proteins and then injected with CoV RBDs26 (12.5 nM–200 nM); blank flow cells were used to correct the binding response. The monovalent binding affinities of the fusion proteins were measured using the BIAcore 2000 system and the surface plasmon resonance (SPR) method.

### Murine models

The *in vivo* experiments were approved by the Institutional Animal Care and Use Committee (IACUC) of the Second Military Medical University. C57BL/6, DBA/2J and CBA/J mice were purchased from the Animal Center of the Second Military Medical University, and mice were housed in a specific pathogen-free barrier facility. Animals were randomly assigned to treatment groups using a computer-generated randomization sequence. Investigators performing injections and outcome assessment were blinded to group allocation. The sample size (n = 10 per group) was determined based on prior literature using the same models, which demonstrated consistent detection of differences in fetal resorption rates. For the pharmacokinetics study, fusion proteins at a dose of 5 mg/kg body weight were injected into the tail vein of 8-week-old C57BL/6 mice, which were further divided into 15 groups, corresponding to days 1–15. ELISA was used to determine the serum concentrations of the fusion proteins. Before the analysis, blood collected from the septum was centrifuged in heparin-containing tubes to remove blood cells and to obtain plasma samples.

For the immunological model of abortion, DBA/2J-mated CBA/J females were randomly divided into different treatment groups. Fusion proteins (20 mg/kg) were administered twice (days 1.5 and 3.5 dpc) via tail vein injection in CBA/J × DBA/2 mice. The day of vaginal plug formation was considered day 0.5 of coitus. On days 1.5 and 3.5 postcoitum (dpc), 20 mg/kg of drug (fusion proteins or control IgG, i. v.) was administered to mice of different groups. Next, the mice were killed at 14 dpc, and the rates of pregnancy and abortion were calculated.

To develop the passive transfer of the Th17 cell model, immune cells were isolated from the spleens of virgin female C57BL/6 mice, and naïve CD4^+^ T cells were obtained by the positive selection of D62L + cells (CD4^+^ CD62L + T Cell Isolation Kit II; Miltenyi Biotec). For Th17 transfer experiments, PD-L1-Fc or control IgG was injected once on day 7.5 dpc. For the control mice, pregnant C57BL/6 females were injected via the tail vein on day 7.5 dpc with 1 × 10^6^ naïve CD4^+^ T cells resuspended in 200 μL of PBS. For the Th17 cell transfer group, Th17 cells were first induced by the incubation of naïve CD4^+^ T cells under specific conditions. The cells were activated with plate-bound anti-CD28 (2 μg/mL; BD Biosciences) and anti-CD3 (5 μg/mL; BD Biosciences) antibodies. Mouse IL-1β (10 ng/mL), IL-6 (20 ng/mL), TGF-β (2 ng/mL), anti-mouse IL-4 (10 μg/mL), and anti-mouse IFN-γ (10 μg/mL), all purchased from R&D Systems, were added to stimulate the differentiation of Th17 cells for 6 days. On day 7.5 dpc, 1 × 10^6^ polarised cells were collected and resuspended in 100 μL of PBS, and each pregnant C57BL/6 female was injected via the tail vein with Th17 cells. Selected mice were also treated with 20 mg/kg of fusion proteins or control IgG (i.v.) on the same day of cell transfer. All the mice were sacrificed at 14 dpc, and the uteri were examined for the number of healthy and resorbing embryos. Different groups of mice were sacrificed by cervical dislocation. On day 14 of pregnancy, the uteri were removed, and the implantation sites were documented. The abortion sites were identified by their small size and necrotic, haemorrhagic appearance. The percentage of abortions was calculated as the ratio of resorption sites to total implantation sites (resorption plus normal implantation sites).

The dose of PD-L1-Fc (20 mg/kg) was selected based on prior studies using PD-L1-Fc fusion proteins in murine immunomodulatory models (Wang et al., 2011; Song et al., 2015), which demonstrated effective systemic exposure without toxicity. The choice of 1 × 10^6^ Th17 cells for adoptive transfer follows previously published protocols for inducing pregnancy loss (Wang et al., 2014), ensuring reproducible Th17-driven pathology.

Embryo resorption was identified based on established macroscopic criteria, including reduced implantation site size, tissue discoloration, and hemorrhagic appearance. Although representative photographs were not collected for this study, all assessments were performed by investigators blinded to treatment group, ensuring reproducibility.

Treatment timeline:

- CBA/J × DBA/2 model: PD-L1-Fc was administered on days 1.5 and 3.5 post-coitum (pre-implantation to early implantation period). Uterine tissues were collected at day 14.
- Th17-transfer model: naïve or Th17 cells were transferred on day 7.5 post-coitum (post-implantation), followed by PD-L1-Fc injection on the same day. Tissues were harvested at day 14.

These timelines are now explicitly described for clarity.

### Anesthesia and euthanasia

All animal experiments were conducted under adequate anesthesia to minimize suffering. For major survival surgeries, mice were anesthetized with 2% isoflurane. The anesthetic was delivered via a precision vaporizer at a flow rate of 1.5 L per minute of oxygen through a nose cone. The depth of anesthesia was monitored by checking for the absence of a pedal withdrawal reflex.

For terminal procedures, euthanasia was carried out using one of the following methods:

1. Intraperitoneal injection of sodium pentobarbital at a dose of 150 mg/kg.
2. CO_2_ inhalation, administered at a fill rate of 30% of the euthanasia chamber volume per minute.

Death was confirmed by the cessation of respiration and the absence of cardiac activity.

### Real-time polymerase chain reaction (PCR)

An RNeasy Mini Kit (Qiagen) was used to isolate total RNA. Using the ABI PRISM 7900HT instrument, real-time quantitative PCR was performed using the commercially available TaqMan probes Mm00439618 (IL-17a), Mm01168134 (IFn-γ), and Mm01288386 (IL-10). β-Actin was selected as the endogenous control, and data were analysed using SDS v2.3 (Applied Biosystems). cDNA synthesis was performed using the PrimeScript RT Reagent Kit (Takara, Japan) following the manufacturer’s instructions.

### Isolation of decidual T cells and flow cytometry analysis

On day 14 of pregnancy, the decidua of mice was collected for T-cell isolation using magnetic beads following the manufacturer’s instructions (MACS; Miltenyi Biotec, Germany). PMA (50 ng/mL, Sigma) and ionomycin (750 ng/mL, Sigma) were added to restimulate T cells for 5 h. Next, staining for the intracellular cytokines CD4, IFN-γ, IL-10 and IL-17A was performed. ELISA kits for IFN-γ, IL-17A, and IL-10 (R&D Systems; cat. nos. DY485, DY421, and DY417) were used according to the manufacturer’s instructions. Detection ranges were 15.6–1,000 pg/mL. All samples were analyzed in duplicates.

### Statistical analysis

Statistical analyses were performed using GraphPad Prism 9.0. Normality was assessed using the Shapiro–Wilk test; two-group comparisons used Student’s t-test, and multi-group analyses used one-way ANOVA with Tukey’s *post hoc* test. Unless otherwise specified, the significant differences between two groups and among three or more groups were evaluated by Student’s t-test and ANOVA, respectively. Differences between samples were considered statistically significant when P < 0.05. Data are presented as mean ± SD unless otherwise stated. Exact biological replicate numbers (n) are indicated in figure legends. Exact p-values are provided when <0.1, and categorical outcomes were assessed using Fisher’s exact test.

## Results

### Fc engineered PD-L1 fusion protein

**FIGURE 1:**
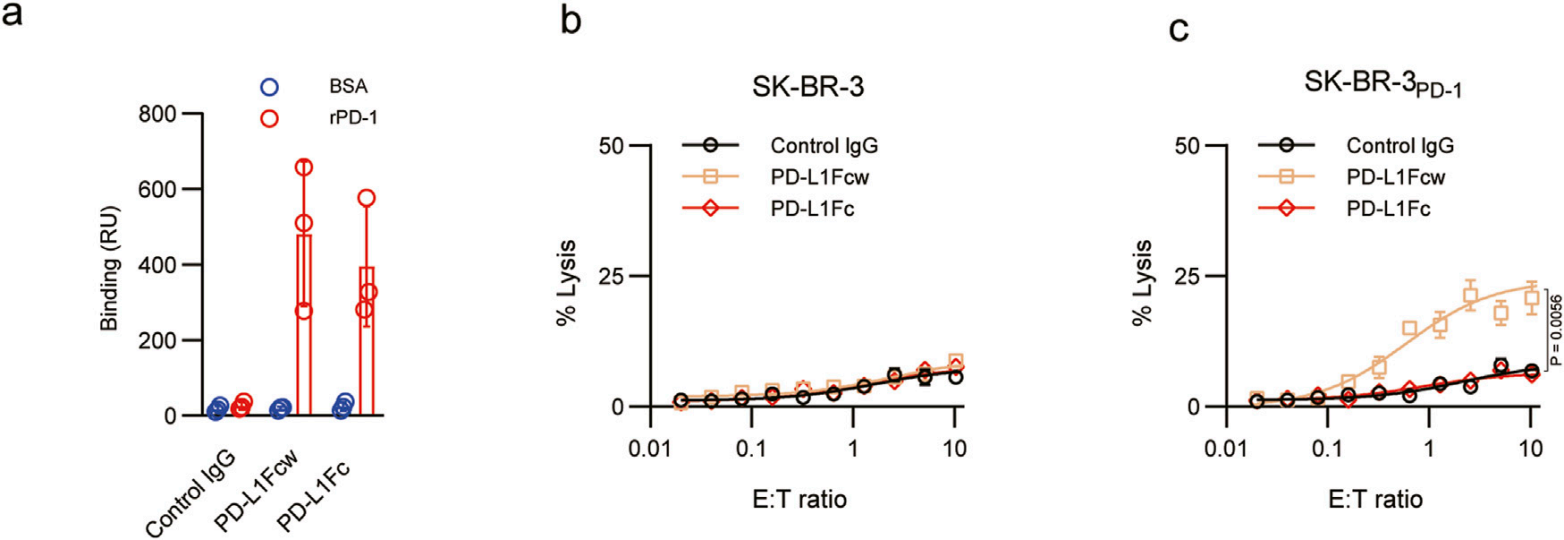
Characterisation of Fc fused PD-L1 protein. **(a)** Binding of the fusion proteins to PD1. The fusion proteins were tested for binding to immobilised PD-1 or BSA using surface plasmon resonance on a BIAcore 2000 instrument. Binding was quantified as an increase in RU at 60 s after the end of injection compared with baseline established 20 s before injection. **(b)** *In vitro* ADCC assay using cell lines that do not express PD-1. **(c)** Statistical comparisons were performed between PD-L1-Fcw vs. control IgG, and PD-L1-Fc vs. control IgG at each E:T ratio.

Fc-fused proteins induce Fc-γ-dependent, antibody-dependent, and cell-mediated cytotoxicity caused by both murine IgG2a and human IgG1. Given this concern, we sought to develop murine fusion proteins with murine IgG Fc LALA-PG variants linked to the extracellular domain of either murine PD-L1 (PD-L1Fc). The LALA-PG Fc variants block not only complement binding and fixation but also cytotoxicity. Fc-engineered PD-L1 fusion proteins showed similar binding affinity to PD-1 compared with wild-type Fc fusion proteins (PD-L1Fcw) (Figure 1a). We evaluated the ability of different Fc-fused proteins to induce ADCC lysis in SK-BR-3 cells and PD-1-expressing SK-BR-3 cells *in vitro* utilising PBMC-dependent ADCC assays (Figures 1b,c). PD-positive cells were resistant to ADCC-mediated lysis by the Fc mutant PD-L1 protein, while the cells are sensitive to wild-type Fc protein over a range of E:T ratios. Therefore, fusion proteins with the LALA-PG mutant were chosen for further experiments in our study.

In *in vitro* biochemistry analysis, PD-L1Fc displayed denaturation temperatures similar to those for the previously reported ACE2-Fc protein, suggesting IgG-like stability. After storage at a concentration of 1 mg/mL at 40 °C for 3 weeks, the formation of only minimal amounts (<1%) of high- and low-molecular-weight products was observed. Additionally, as listed in Table 1, the main pharmacokinetic (PK) parameters of PD-L1Fc in mice were very similar to those of PD-L1Fcw and ACE2 and showed that PD-L1Fc had comparable PK properties to those of a conventional IgG molecule (Table 1).

**TABLE 1:** Selected analytical data and pharmacokinetic parameters of recombinant fusion proteins in mice.

Parameter^a^	PD-L1Fcw	PD-L1Fc	ACE2-Fc
HMW formation after storage (% SEC area)^b^	<0.1	<0.1	<0.1
LMW formation after storage (% SEC area)^b^	<0.1	<0.1	<0.1
AUC (day μg mL^−1^)	492.18	538.99	529.84
*T*_*1/2*_ (day)	4.32	5.22	6.66
CL (mL day^−1^ kg^−1^)	8.53	7.40	7.09
VSS (mL kg^−1^)	69.13	67.57	73.17

^a^ Pharmacokinetic parameters were calculated using a noncompartmental analysis. AUC, area under the concentration versus time curve; t1/2, half-life; CL, clearance; VSS, steady-state volume of distribution.

^b^ Quiescent storage for 3 weeks, 40 °C, 1 mg/mL.

### PD-L1-Fc treatment ameliorates abortion rates in abortion prone animals

To explore the therapeutic efficiency of PD-L1-Fc in an experimental animal model, well-established abortion-prone (AP) DBA ⁄ 2J mated CBA ⁄ J mice were treated with PBS, negative control IgG or PD-L1-Fc. As expected, the abortion rates of AP mice were much higher than those of control mice. By contrast, the percentage of foetal rejection in AP mice was significantly ameliorated by PD-L1-Fc administration. This effect was dependent on PD1 because combined therapy of PD-L1-Fc with PD1-Fc completely abolished the protective effect, as shown by an even higher median abortion rate (30.56%). The abortion and implantation rates in each treatment group are summarised in Table 2. In the DBA/2J × CBA/J model, PD-L1-Fc reduced the fetal resorption rate to 8.16% ± SD (Table 2), compared with 25.0% in the control IgG group. The baseline resorption rates observed in saline-treated CBA/J × DBA/2 mice (21.2%) and in Th17-cell-transferred C57BL/6 mice (56.25%) matched previously reported ranges for these established models, confirming their expected pathological behavior prior to PD-L1-Fc intervention. PD-L1-Fc affects the differentiation of uterine T helper cells.

**TABLE 2:** Comparison of fetal resorption rates in pregnant CBA/J mice.

Group	Number	Surviving fetuses	Resorbed fetuses	Resorption rate (%)
Group 1 (CBA/J × BALB/c received saline)	10	68	3	4.2*
Group 2 (CBA/J × DBA/2 received saline)	10	52	14	21.2
Group 3 (CBA/J × DBA/2 received control IgG)	10	48	16	25.0
Group 4 (CBA/J × DBA/2 received PD-L1Fc)	10	45	4	8.16*
Group 5 (CBA/J × DBA/2 received PD-L1Fc + PD1Fc)	10	50	22	30.56

Fisher’s exact test, *P < 0.05 versus Group 2.

Data represent raw counts and percentages with 95% confidence intervals. Primary endpoint: fetal resorption rate. P-values calculated using Fisher’s exact test.

**FIGURE 2:**
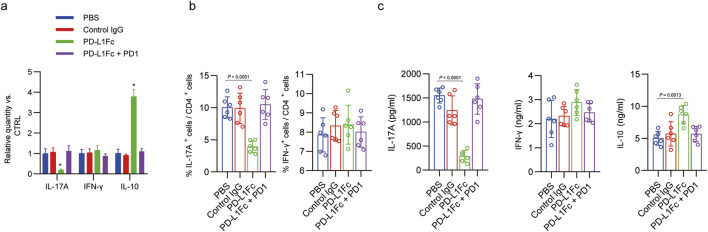
PD-L1-Fc treatment affects the differentiation of uterine T helper cells in mice. **(a)** qPCR of the expression of IL-17a, IFn-γ, and Il-10 mRNA in the different groups. *P* values were calculated from one-way ANOVA followed by Tukey’s *post hoc* test. *P < 0.05. **(b)** The frequencies of CD4^+^ T cells producing IFN-γ or IL-17A in the decidua were determined by flow cytometry after *in vitro* restimulation with PMA and ionomycin. The percentages of IFN-γ+ and IL-17A + T cells are presented as the means ± s.d. of 4–6 individual mice per group. Flow cytometry gating was performed using a standardized workflow (lymphocyte → singlets → CD4^+^ → cytokine^+^). Although representative plots were not captured for publication in this study, all analyses were conducted using identical gating parameters across groups and validated by two independent investigators. **(c)** After restimulation of sorted CD4 T cells (5 × 10^4^) with anti-CD3/CD28 monoclonal antibodies (mAbs) for 24 h, the levels of IFN-γ, IL-17A or IL-10 in the cell culture supernatants were determined using ELISA. *p < 0.05; **p < 0.01 by one-way analysis of variance followed by Bonferroni correction. The data are representative of three independent experiments.

IL-17 is thought to be directly linked to PD-L1 signalling in foetomaternal tolerance. Therefore, we investigated the correlation between PD-L1-Fc treatment and the relative expression of IL-17A, IFN-γ and IL-10 in the murine decidua (Figure 2a). Compared with the control IgG group, the mRNA levels of IL-17 and IL-10 in the group treated with PD-L1Fc were notably modulated; the former was downregulated, and the latter was upregulated (P < 0.05). Moreover, the level of IFN-γ following PD-L1Fc administration was upregulated, albeit not significantly. We further investigated the frequency of Treg cells in the mouse uterus. In contrast to control IgG, in the group administered PD-L1-Fc, the percentage of CD4^+^ T cells producing IL-17A in the uterine cells derived from mating mice was markedly reduced, and the percentage of IFN-γ-producing CD4^+^ T cells was not changed otherwise (Figure 2b). This result indicates the inhibitory effect of PD-L1-Fc on Th17 responses during mouse pregnancy. Likewise, anti-CD3 and anti-CD28 mAbs restimulating uterine CD4^+^ T cells sorted from PD-L1-Fc-treated mice decreased the secretion of IL-17A but increased the secretion of IL-10 (Figure 2c), consistent with a previous report that the interaction of PD-L1 with PD-1 expressed on T cells prohibits the development of Th17 cells and alleviates the Th17 cell-mediated immune response.

### PD-L1-Fc showed a protective effect in a Th17 cell transfer-induced mouse abortion model

To further determine the role of Th17 cells in PD-L1Fc treatment, a Th17 cell-based abortion model was adopted in our study. Consistent with a previous report, pregnant mice adoptively transferred with Th17 cells at gd 7.5 had significant foetal loss compared with those adoptively transferred with naïve T cells. Conversely, the absorption rate was notably increased in the mice treated with PD-L1Fc, suggesting that PD-L1Fc treatment combated Th17 cell-induced foetal loss. In the Th17 transfer model, PD-L1-Fc significantly reduced fetal resorption compared with control IgG (Table 3).

**TABLE 3:** Comparison of fetal resorption rates in pregnant C57BL/6 mice.

Group	Number	Surviving fetuses	Resorbed fetuses	Resorption rate (%)
Group 1 (C57BL/6 mice transfer naïve CD4^+^ T cells)	10	62	2	3.125*
Group 2 (C57BL/6 mice transfer Th17 T cells)	10	28	36	56.25
Group 3 (C57BL/6 mice transfer Th17 T cells + control IgG)	10	19	29	60.42
Group 4 (C57BL/6 mice transfer Th17 T cells + PD-L1Fc)	10	41	9	18.0*
Group 5 (C57BL/6 mice transfer Th17 T cells + PD1Fc)	10	22	30	57.59

Fisher’s exact test, *P < 0.05 versus Group 2.

Data represent raw counts and percentages with 95% confidence intervals. Primary endpoint: fetal resorption rate. P-values calculated using Fisher’s exact test.

## Discussion

PD-L1 signalling has been demonstrated to be vital for the induction of immune tolerance and is a promising target of autoimmune disease therapy. The binding of PD-L1 with its receptor PD-1 on T cells mediates programmed cell death and inhibits cytokine secretion, leading to the downregulation of T-cell responses (Butte et al., 2007). The PD-1:PD-L1 pathway is interesting in the context of maternal immune tolerance to foetal antigens because balanced immune responses are essential to maintain a successful pregnancy. In this report, we demonstrate that the Fc-fused PD-L1 protein reduces the foetal abortion rate in abortion-prone animal models. Recombinant Fc fusion, which can extend the plasma residence time of soluble proteins, improve *in vivo* efficacy, and gain immunoreactive functions, has been widely used in modern biopharmaceuticals. To further minimise these side effects of IgG-like molecules, we engineered the Fc portion with LALA-PG mutations and tested it in an *in vitro* ADCC assay. The PD-L1Fc protein with engineered Fc showed negligible cytotoxic effects on PD-1-expressing cells. In addition to restraining Th17 differentiation, PD-L1-Fc may exert broader immunomodulatory effects. Engagement of PD-1 on decidual T cells can reduce activation, modulate APC function, and shift cytokine production toward a tolerogenic IL-10–rich milieu. Although our study focused on Th17/Treg dynamics, PD-L1–mediated inhibition of antigen-presenting cell maturation and local inflammatory pathways likely contributes to the observed protection.

Notably, the PD-L1:PD-1 interaction inhibited the differentiation of Th17 cells. The engagement of PD-1 expressed on IL-27-primed CD4^+^ T cells and mesenchymal stem cells (MSCs) with PD-L1 selectively suppresses the induction of Th17 cells (Hirahara et al., 2012; Luz-Crawford et al., 2012). Under Th17 cell-polarising conditions, IL-17 production by CD4^+^ T cells was notably suppressed by the soluble PD-L1 fusion protein, while the inhibition of mature Th17 cells by MSCs was reversed by the anti-PD-L1 neutralising antibody. Additionally, PDL1 blockade induces the apoptosis of Tregs and a shift towards a higher frequency of Th17 cells, breaking foetomaternal tolerance in mouse models (D’Addio et al., 2011). This finding is consistent with our observation that treatment of DBA/2J × CBA/J mice with PD-L1-Fc leads to a preferential suppression of the expression of IL-17a and expansion of Th17 cells in the decidua. Previous interventions targeting PD-1/PD-L1 in reproductive models have primarily used blocking antibodies or genetic deficiency, both of which exacerbate fetal loss. In contrast, PD-L1-Fc provides transient, ligand-driven signaling without immune-cell depletion, suggesting a mechanistically distinct and potentially safer immunomodulatory approach. Additionally, we showed a direct relationship between PD-L1Fc treatment and TH17 cell-induced foetal loss in mouse models. Th17 cells are considered a critical lineage of proinflammatory T helper cells involved in autoimmune disease development (Korn et al., 2009; Park et al., 2005; Wang et al., 2010) and impede the induction of tolerance in transplantation and pregnancy (D’Addio et al., 2011; Yuan et al., 2008). Moreover, Th17 cells also play a significant role in inflammatory infiltration in patients with recurrent spontaneous abortions (Wang et al., 2010). Redundant Th17 cells directly caused foetal loss in an *in vivo* mouse model in a previous report (Wang et al., 2014). In our study, this effect could be abolished with PD-L1Fc treatment, suggesting that pregnancy pathologies induced by Th17 cells may be affected by treatments with PD-L1Fc. Wild-type PD-L1-Fc (PD-L1-Fcw) induces ADCC in PD-1–expressing cells (Figure 1), which raises safety concerns during pregnancy. For ethical reasons, and given our study’s focus on evaluating a non-cytolytic therapeutic candidate, PD-L1-Fcw was not administered *in vivo*.

Mechanistically, the observed reduction in Il17a expression together with increased Il10 suggests that PD-L1-Fc modulates the Th17/Treg axis in a manner highly relevant to fetomaternal tolerance. Unlike autoimmune models in which both effector and regulatory T cells expand simultaneously, the pregnant uterus is uniquely dependent on controlled Th17 restraint. The Fc-silenced PD-L1-Fc used here offers a potential translational advantage by avoiding immune-cell depletion while selectively attenuating Th17-driven inflammation.

This study has several limitations. First, murine PD-L1 and gestational immunology differ from humans, and translational extrapolation requires caution. Second, while LALA-PG engineering reduces cytotoxicity, long-term safety and potential off-target immunosuppressive effects remain to be evaluated. Third, we cannot exclude subtle effects of PD-L1-Fc on implantation biology independent of immune modulation.

In conclusion, we found that treatment with PD-L1-Fc during pregnancy exerts a protective effect on embryo resorption by promoting the regulation of decidual CD4^+^ T cells. Unlike earlier studies employing wild-type PD-L1-Fc in autoimmune disease, our work demonstrates that an Fc-engineered (LALA-PG) PD-L1 fusion protein effectively suppresses Th17-driven pathology in pregnancy while avoiding Fc-mediated cytotoxicity. This safety-oriented modification is particularly relevant for pregnancy, where unintended depletion of PD-1–expressing immune cells could be harmful. A limitation of the study is the absence of stored representative dot plots; however, the quantitative data were derived from rigorously standardized gating applied uniformly across biological replicates. Moreover, PD-L1-Fc effectively ameliorated the abortion rate not only in a DBA/2J × CBA/J mouse model but also in a Th17 cell-induced abortion model, suggesting that PD-L1-Fc is a potential therapeutic agent for treating human spontaneous abortion. Given the association of elevated Th17 responses with recurrent spontaneous abortion in humans, PD-L1-Fc may represent a promising therapeutic candidate for immune-mediated pregnancy loss. Its transient activity, preserved PD-1 specificity, and lack of cytotoxic Fc effector function support its suitability for selective immunomodulation during early gestation. Challenges for translation include pharmacokinetic optimization, species-specific PD-1 interactions, and rigorous safety assessment in pregnancy. Therefore, PD-L1-Fc treatment may be a novel and promising biological strategy to inhibit inflammatory symptoms associated with the immune-pathogenic type of spontaneous abortion.

## Data Availability

The original contributions presented in the study are included in the article/supplementary material, further inquiries can be directed to the corresponding authors.

## References

[B1] Butte M. J. Keir M. E. Phamduy T. B. Sharpe A. H. Freeman G. (2007). Programmed death-1 ligand 1 interacts specifically with the B7-1 costimulatory molecule to inhibit T cell responses. Immunity 27 (1), 111–122. 10.1016/j.immuni.2007.05.016 17629517 PMC2707944

[B2] D’Addio F. Riella L. V. Mfarrej B. G. Chabtini L. Adams L. T. Yeung M. (2011). The link between the PDL1 costimulatory pathway and Th17 in fetomaternal tolerance. J. Immunol. 187 (9), 4530–4541. 10.4049/jimmunol.1002031 21949023 PMC3197965

[B3] Freeman G. J. Long A. J. Iwai Y. Bourque K. Chernova T. Nishimura H. (2000). Engagement of the PD-1 immunoinhibitory receptor by a novel B7 family member leads to negative regulation of lymphocyte activation. J. Exp. Med. 192 (7), 1027–1034. 10.1084/jem.192.7.1027 11015443 PMC2193311

[B4] Guleria I. Khosroshahi A. Ansari M. J. Habicht A. Azuma M. Yagita H. (2005). A critical role for the programmed death ligand 1 in fetomaternal tolerance. J. Exp. Med. 202 (2), 231–237. 10.1084/jem.20050019 16027236 PMC2213002

[B5] Hirahara K. Ghoreschi K. Yang X.-P. Takahashi H. Laurence A. Vahedi G. (2012). Interleukin-27 priming of T cells controls IL-17 production in trans via induction of the ligand PD-L1. Immunity 36 (6), 1017–1030. 10.1016/j.immuni.2012.03.024 22726954 PMC3785111

[B6] Holets L. M. Hunt J. S. Petroff M. G. (2006). Trophoblast CD274 (B7-H1) is differentially expressed across gestation: influence of oxygen concentration. Biol. Reprod. 74 (2), 352–358. 10.1095/biolreprod.105.046581 16251499

[B7] Jafari R. M Zolbanin N. Rafatpanah H. Majidi J. Kazemi T. J. (2017). Fc-fusion proteins in therapy: an updated view. Curr. Med. Chem. 24 (12), 1228–1237. 10.2174/0929867324666170113112759 28088904

[B8] Keir M. E. Francisco L. M. Sharpe A. (2007). PD-1 and its ligands in T-cell immunity. Curr. Opin. Immunol. 19 (3), 309–314. 10.1016/j.coi.2007.04.012 17433872

[B9] Korn T. Bettelli E. Oukka M. Kuchroo V. K. (2009). IL-17 and Th17 cells. Annu. Rev. Immunol. 27, 485–517. 10.1146/annurev.immunol.021908.132710 19132915

[B10] Luz-Crawford P. Noël D. Fernandez X. Khoury M. Figueroa F. Carrión F. (2012). Mesenchymal stem cells repress Th17 molecular program through the PD-1 pathway. PLoS One 7 (9), e45272. 10.1371/journal.pone.0045272 23028899 PMC3444478

[B11] Nishimura H. Nose M. Hiai H. Minato N. Honjo T. (1999). Development of lupus-like autoimmune diseases by disruption of the PD-1 gene encoding an ITIM motif-carrying immunoreceptor. Immunity 11 (2), 141–151. 10.1016/s1074-7613(00)80089-8 10485649

[B12] Nishimura H. Okazaki T. Tanaka Y. Nakatani K. Hara M. Matsumori A. (2001). Autoimmune dilated cardiomyopathy in PD-1 receptor-deficient mice. Science (1979). 291 (5502), 319–322. 10.1126/science.291.5502.319 11209085

[B13] Okuyama M. Mezawa H. Kawai T. Urashima M. J. (2019). Elevated soluble PD-L1 in pregnant women's serum suppresses the immune reaction. Front. Immunol. 10, 86. 10.3389/fimmu.2019.00086 30833943 PMC6387906

[B14] Park H. Li Z. Yang X. O. Chang S. H. Nurieva R. Wang Y.-H. (2005). A distinct lineage of CD4 T cells regulates tissue inflammation by producing interleukin 17. Nat. Immunol. 6 (11), 1133–1141. 10.1038/ni1261 16200068 PMC1618871

[B15] Petroff M. G. Chen L. Phillips T. A. Azzola D. Sedlmayr P. Hunt J. (2003). B7 family molecules are favorably positioned at the human maternal-fetal interface. Biol. Reprod. 68 (5), 1496–1504. 10.1095/biolreprod.102.010058 12606489

[B16] Song M.-Y. Hong C.-P. Park S. J. Kim J.-H. Yang B.-G. Park Y. (2015). Protective effects of Fc-fused PD-L1 on two different animal models of colitis. Gut 64 (2), 260–271. 10.1136/gutjnl-2014-307311 24902766

[B17] Veras E. Kurman R. J. Wang T.-L. Shih I.-M. (2017). PD-L1 expression in human placentas and gestational trophoblastic diseases. Int. J. Gynecol. Pathol. 36 (2), 146–153. 10.1097/PGP.0000000000000305 27362903 PMC5518625

[B18] Wang W.-J. Hao C.-F. Yin G.-J. Bao S.-H. Qiu L.-H. Lin Q.-D. (2010). Increased prevalence of T helper 17 (Th17) cells in peripheral blood and decidua in unexplained recurrent spontaneous abortion patients. J Reprod Immunol. 84 (2), 164–170. 20106535 10.1016/j.jri.2009.12.003

[B19] Wang G. Hu P. Yang J. Shen G. Wu X. (2011). The effects of PDL-Ig on collagen-induced arthritis. Rheumatol. Int. 31 (4), 513–519. 10.1007/s00296-009-1249-0 20035333

[B20] Wang W.-J. Liu F.-J. Hao C.-F. Bao H.-C. Qu Q.-L. Liu X.-M. (2014). Adoptive transfer of pregnancy-induced CD4+ CD25+ regulatory T cells reverses the increase in abortion rate caused by interleukin 17 in the CBA/J× BALB/c mouse model. Hum Reprod. 29 (5), 946–952. 24556316 10.1093/humrep/deu014

[B21] Yuan X. Paez-Cortez J. Schmitt-Knosalla I. D'Addio F. Mfarrej B. Donnarumma M. (2008). A novel role of CD4 Th17 cells in mediating cardiac allograft rejection and vasculopathy. J. Exp. Med. 205 (13), 3133–3144. 10.1084/jem.20081937 19047438 PMC2605226

